# Self-assembled targeted nanoparticles based on transferrin-modified eight-arm-polyethylene glycol–dihydroartemisinin conjugate

**DOI:** 10.1038/srep29461

**Published:** 2016-07-05

**Authors:** Kefeng Liu, Lin Dai, Chunxiao Li, Jing Liu, Luying Wang, Jiandu Lei

**Affiliations:** 1 Beijing Key Laboratory of Lignocellulosic Chemistry, Beijing Forestry University, Beijing 100083, P. R. China; 2 Tianjin Key Laboratory of Pulp & Paper, Tianjin University of Science & Technology, Tianjin 300457, P. R. China

## Abstract

Poor delivery of insoluble anticancer drugs has so far precluded their clinical application. In this study, an efficient tumor targeted-nanoparticle delivery system, transferrin-eight-arm-polyethylene glycol–dihydroartemisinin nanoparticles (TF-8arm-PEG-DHA NPs) for the vehiculation of dihydroartemisinin (DHA) was first prepared and evaluated for its targeting efficiency and cytotoxicity *in vitro* and *in vivo* to Lewis lung carcinoma (LLC) cells, which overexpress transferrin receptors (TFRs). The synthesized TF-8arm-PEG–DHA NPs had high solubility (~102 fold of free DHA), relatively high drug loading (~10 wt% DHA), long circulating half-life and moderate particle size (~147 nm). The *in vitro* cytotoxicity and *in vivo* tumor growth inhibition studies in LLC-tumor bearing mice confirmed the enhanced efficacy of TF-modified 8arm-PEG-DHA NPs compared to free DHA and non-modified 8arm-PEG-DHA NPs. All these results together supported that the formulation developed in this work exhibited great potential as an effective tumor targeting delivery system for insoluble anticancer drugs.

The efficacy of cancer treatment using chemotherapy with many small molecule anti-cancer drugs is usually limited due to poor solubility, short circulating half-life, low bioavailability and the toxicity to normal tissues^1^.

Artemisinin is an active ingredient derived from traditional Chinese medicine Artemisia annua L. (commonly known as Annual Wormwood) and contains an endoperoxide bridge structure^2^,^3^. Recent studies have indicated that artemisinin and its derivates can inhibit the growth of a variety of human tumor cell lines and demonstrate selective cytotoxicity to tumor cells. The anti-tumor effects of artemisinin have been proposed to be anti-angiogenesis and modulation of the expression of tumor-related genes^4^,^5^. Dihydroartemisinin (DHA) is the most potent of artemisinin derivatives. It has been found that DHA has antiproliferative effects on various tumor cell lines including cancers of the lung, breast, colon, pancreas, liver, ovary and prostate^6^,^7^. Although DHA has demonstrated an anticancer activity, it still has some drawbacks that limited its clinical appilication, such as low bioavailability, caused by its poor solubility in solution and blood, an initial burst release effect, and high peak plasma concentration from its rapid metabolism^8^,^9^,^10^.

To overcome above-mentioned problems, many vehicles, such as hydrogels, liposomes, dendrimers, prodrugs, and polymeric nanoparticles, have been investigated by researchers^11^,^12^,^13^. Of all these methods, the use of polymer nanoparticles has become one of the most exciting carriers to deliver insoluble anticancer drugs. The polymer nanoparticles possess better pharmacological profiles for the accumulation in tumor sites due to the enhanced permeability and retention (EPR) effect, which resulted in the decrease in adverse effects and improvement of drug tolerance^14^,^15^. Various bioavailable polymers have been selected to overcome the disadvantages of the insoluble anti-cancer drug. Linear polyethylene glycol (PEG) is the most widely used for the development of drugs because of its high solubility in aqueous solution, simple end-group modification, non-toxic, non-immunogenic, and nonantigenic^16^. However, a traditional linear PEG used in the drug delivery system has only one or two active sites available, which results in the poor loading capacity of small molecule drugs^17^. In addition, some studies show that the PEG with a comparatively low molecular weight may face the risk of being fast removed by kidney owe to its small size^18^,^19^. Therefore, multiple PEG with more functional groups and appropriate molecular weight (10 kDa) is endowed with the ability to ensure the high drug loading and the greatest drug enhancements.

It may be possible for nanoparticles to targeted delivery of drug to specific cells by attachment of a specific ligand, which recognizes its receptor on target cells. A lot of ligands have been investigated to date, including antibodies^20^,^21^, aptamers^22^,^23^, peptides^24^, sugar moieties^25^, folate residues^26^,^27^ and transferrin^28^,^29^,^30^.

Transferrin (TF, MW 80 kDa) is a serum glycoprotein that helps to transport iron required as a cofactor for DNA synthesis into rapidly growing cells via the transferrin receptor (TFR)^31^. Because of the rapid rate of proliferation of cancer cells, their demand for iron is much greater than normal cells. This leads to an increased expression of TFR on the surface of cancer cells that can be exploited for the purpose of active targeting of nanocarriers to these cells. TF-conjugated nanoparticles can selectively deliver anticancer drugs to tumor cells overexpressing TFR through TFR-mediated endocytosis^32^.

In this study, 8arm-PEG-DHA NPs were prepared to provide enhanced solubility and stability in aqueous solution. In addition, transferrin (TF), the targeting ligand, was conjugated to the side chain of 8arm-PEG-DHA NPs for target ability. This targeting delivery system can provide lower systemic toxicity and higher therapeutics efficiency. This paper reports the design, synthesis, *in vitro* and *in vivo* evaluation of TF-8arm-PEG-DHA NPs with TF as the targeting ligand to enhance the cellular uptake and anti-cancer activity through receptor-mediated endocytosis between TF and TFR.

## Results

### Synthesis of 8arm-PEG–DHA and TF-8arm-PEG–DHA conjugates

**Figure 1:**
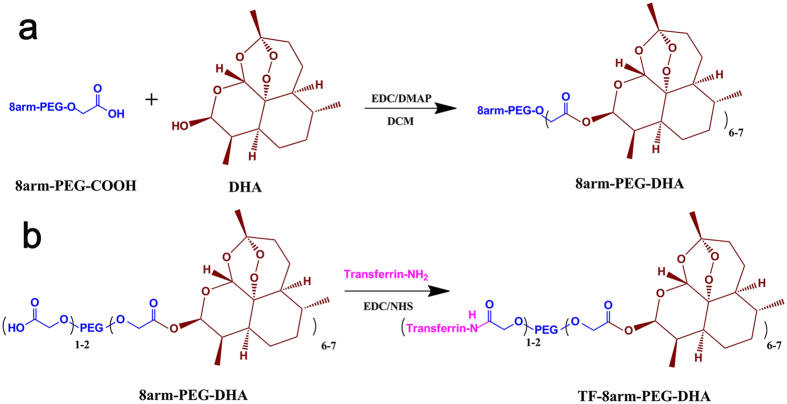
Synthesis of 8arm-PEG–DHA and TF-8arm-PEG–DHA conjugates.

Linear PEG is the most widely used nonionic polymer because of its high aqueous solubility in the field of polymer-based drug delivery, but it is limited by its low drug-binding capacity. Here, 8arm-PEG-COOH was selected due to its high drug-loading capacity compared with linear PEG. The 8arm-PEG–DHA was synthesized by an esterification reaction between the carboxyl group of 8arm-PEG-COOH and the hydroxyl group of DHA. In addition, the targeting ligand, TF, was introduced to 8arm-PEG–DHA conjugate via a coupling reaction (Fig. 1a,b).

### Characterization of 8arm-PEG–DHA and TF-8arm-PEG–DHA conjugate

**Figure 2:**
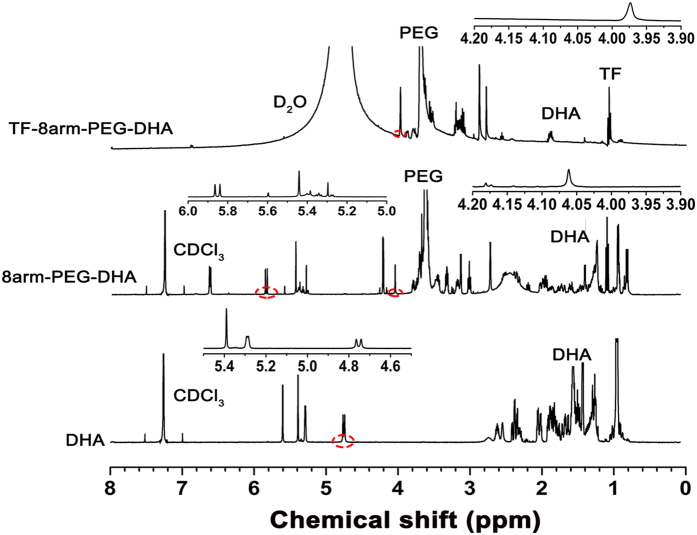
^1^H-NMR spectra of DHA in CDCl_3_ (**a**), 8arm-PEG–DHA in CDCl_3_ (**b**), TF-8arm-PEG–DHA in D_2_O (**c**).

Figure 2a–c show the ^1^H-NMR spectra of DHA, 8arm-PEG–DHA and TF-8arm-PEG–DHA, where the signals at 0.80–2.90 are attributed to the most characteristic peak protons of DHA^33^, those at 3.40–3.85 (4 nH, –(CH2CH2O)n–) and 4.20 (2H, –CH2OC(O)O–) to the methylene protons of PEG, and those at 0.81–1.30 are attributed to the proton peaks of TF^34^. The doublet around 4.78 (1H, CH) of DHA moving to 5.82 (1H, CH) in 8arm-PEG-DHA spectra indicated successful synthesis of ester bonds between PEG and DHA. In addition, the proton peaks of TF and 8arm-PEG-DHA were chemically shifted due to the steric effect. CH2 (non-repeat molecular of PEG-DHA) was shifted from 4.06 to 3.97 because the macromolecule as a TF was introduced to the carboxyl group of PEG in 8arm-PEG-DHA.

Loading content of nanoparticle is an important factor to be considered for researches *in vitro* or *in vivo*. Compared to the traditional linear PEG, the drug-loading capacity was obvious increased due to the 8arm-PEG was selected. The mass ratio of 8arm-PEG-DHA NPs and TF-8arm-PEG-DHA NPs calculated by HPLC were 15.12% and 10.39%, and the molar ratio was 6.26 ± 0.52, 93.39 ± 7.56, respectively (Table 1). For the determination a bradford protein assay with pure holo-transferrin as standard was applied for the determination of the average amount of TF that conjugated to the 8arm-PEG-DHA^35^. The calculated coupling efficiency was 1 mg TF/2 mg 8arm-PEG-DHA conjugate.

### Nanoparticle formulation

**Figure 3:**
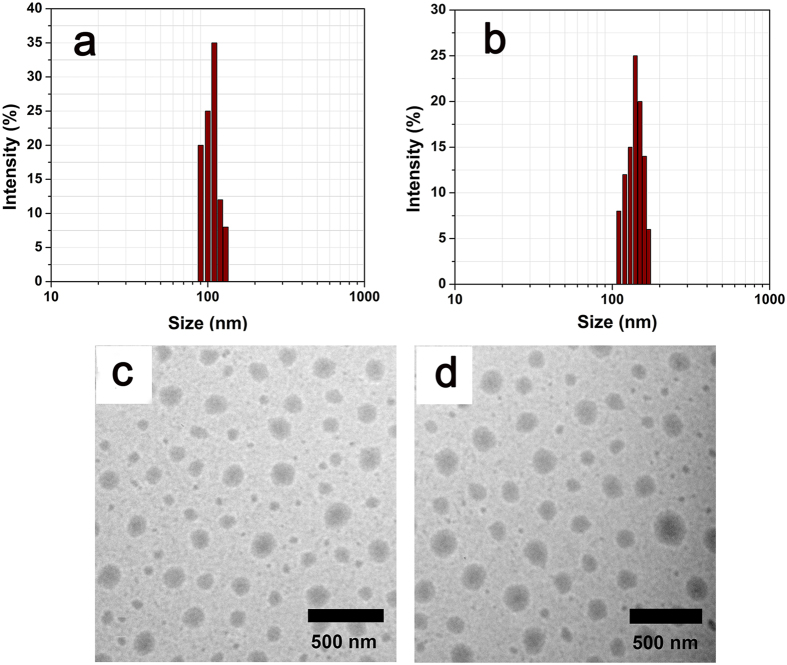
Particle size and morphological observation of nanoparticles. Particle size of 8arm-PEG-DHA NPs (**a**) and TF-8arm-PEG-DHA NPs (**b**) analyzed by DLS. TEM of 8arm-PEG-DHA NPs (**c**) and TF-8arm-PEG-DHA NPs (**d**).

The synthesized polymeric conjugates, such as 8arm-PEG-DHA and TF-8arm-PEG-DHA, have amphiphilic characteristics due to the hydrophilic portion as a PEG and hydrophobic portion as a DHA. These amphiphilic characteristics can form nanoparticles by self-aggregation and consisted of a hydrophobic core and hydrophilic shell in an aqueous environment. As shown in Fig. 3, the particle size distribution and morphology of 8arm-PEG-DHA NPs (Fig. 3a,c) and TF-8arm-PEG-DHA NPs (Fig. 3b,d) were indicated. The particle size distribution and morphology were measured by dynamic light scattering (DLS) and TEM. 8arm-PEG-DHA NPs and TF-8arm-PEG-DHA NPs showed a unimodal and narrow particle size distribution (Fig. 3a,b), and had a spherical shape. The particle size was lists in Table 1. When TF was conjugated to 8arm-PEG-DHA, the particle size was increased from 112.42 ± 17.28 nm to 147.64 ± 21.36 nm. The increasing particle size of the nanoparticles was observed by both DLS and TEM.

### Solubility test against aqueous solution

**Figure 4:**
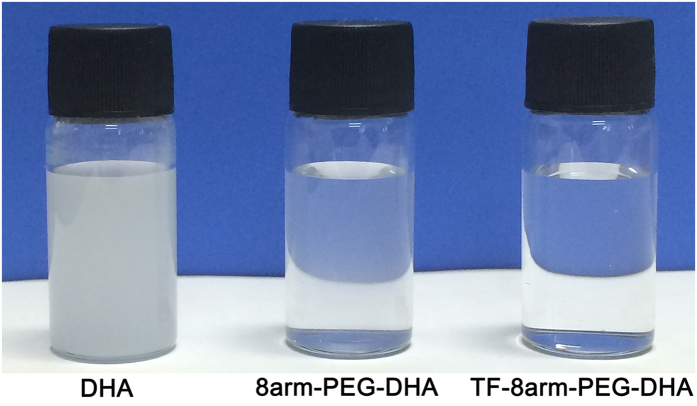
Solubility photography of DHA, 8arm-PEG–DHA NPs and TF-8arm-PEG–DHA NPs against aqueous solution and transmittance was measured using UV method.

Generally, DHA has poor solubility in aqueous solutions; the solubility of DHA in water is less than 0.1 mg/mL^36^. Therefore, the use of DHA can cause side effects due to the excipient, such as Cremophor EL (CrmEL). In this study, to overcome the poor solubility of DHA in aqueous solutions, the polymeric nanoparticles were prepared with PEG and TF, which are hydrophilic molecules. This resulted in an obvious increase in the solubility of DHA. The concentration of each sample (DHA, 8arm-PEG-DHA NPs, and TF-8arm-PEG-DHA NPs) was based on the amount of that DHA (final DHA concentration: 10 mg/mL). The transmittances of DHA, 8arm-PEG-DHA NPs, and TF-8arm-PEG-DHA NPs were 0.95%, 90.24%, and 96.70% at 210 nm, respectively (Table 1). As shown in Fig. 4, the hydrophilic molecular-introduced 8arm-PEG-DHA and TF-8arm-PEG-DHA NPs showed a significant increase in solubility whereas the free DHA was opaque. In addition, TF-conjugated TF-8arm-PEG-DHA NPs showed higher solubility than the other samples, such as DHA and 8arm-PEG-DHA NPs. This shows that the hydrophilicity of DHA was increased due to the introduction of hydrophilic molecules, indicating that the prepared novel nanoparticles can obvious increase the solubility of DHA.

### *In vitro* drug relaease

**Figure 5:**
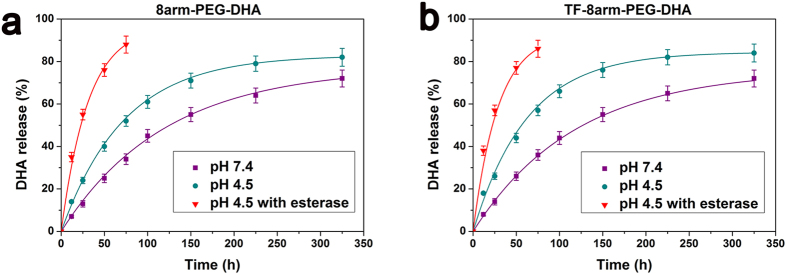
Drug release kinetics in PBS at 37 °C from the 8arm-PEG–DHA NPs (**a**) and TF-8arm-PEG–DHA NPs (**b**).

The release of DHA from nanoparticles in PBS was analyzed to affirm that hydrolysis of the ester bonds would occur. The nanoparticles were first placed in PBS solutions to simulated biological fluids, and the rates of hydrolysis were measured by HPLC analysis. The stability of the nanoparticles was determined in PBS solutions at pH 7.4 and pH 4.5 to simulate the cytoplasmic/extracellular space and the lysosome, respectively. As shown in Fig. 5a,b, 8arm-PEG–DHA NPs and TF-8arm-PEG–DHA NPs very slowly hydrolyzed and released the DHA at a weakly acidic or neutral pH without the common burst release phenomenon. However, the hydrolyzed DHA displayed a preferential release under weakly acidic conditions (pH 4.5). This accelerated release under acidic conditions was attributed to the re-protonation of the hydroxyl group of DHA. Providing the route that NPs after transferrin modification, they would be typically fuses to an early endosome, and then be trapped in lysosomal after cellular uptake through transferrin receptor-mediated endocytosis, the acid-facilitated release can be a benefit to the enhanced cytotoxicity^32^,^37^,^38^. However, in the presence of esterase, which is abundant in cytoplasm, all the nanoparticles quickly hydrolyzed and released the DHA. The resulting hydrolysis data appear in Fig. 5. The curves in Fig. 5 represent best first-order fits to the data, and the half-lives for these curves are given in Table 1.

### *In vitro* cell cytotoxicity

**Figure 6:**
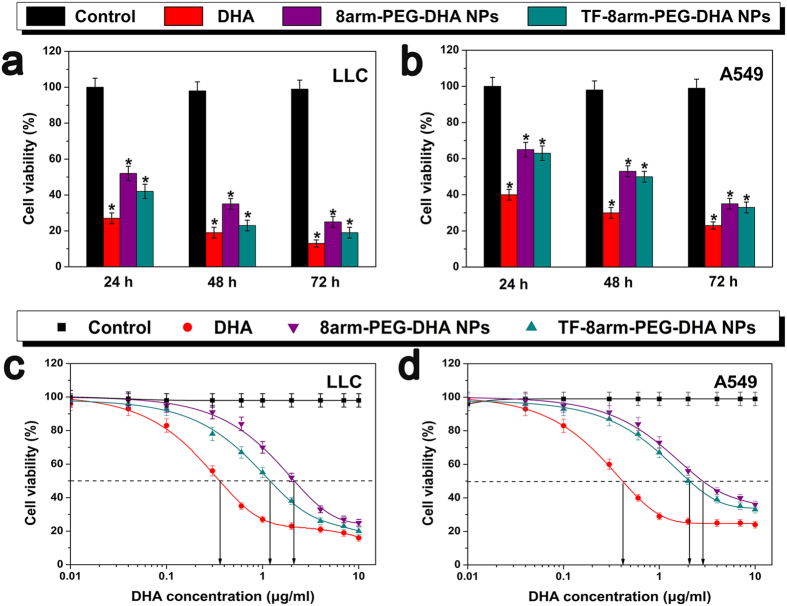
Cell viability of LLC (**a**) and A549 (**b**) cells treated with 10 μg/mL of DHA, 8arm-PEG-DHA NPs and TF-8arm-PEG-DHA NPs (equivalent to free DHA) was measured by CCK-8 assay (n = 3, error bars represent standard deviation). CCK-8 assay of DHA, 8arm-PEG-DHA NPs and TF-8arm-PEG-DHA NPs with different concentrations in LLC (**c**) and A549 (d) cell lines (n = 3, error bars represent standard deviation).

Once a method of measuring free DHA was established for drug released from the newly synthesized nanoparticles, it was necessary to confirm that the liberated drug maintained its cytotoxic activity. To examine the cytotoxicity of DHA and the nanoparticles, a CCK-8 assay was conducted after incubating cells treated with different formulations. The response of LLC and A549 cells were tested *in vitro* by seeding the cells and exposing them to various concentrations of PBS, free DHA, 8arm-PEG-DHA NPs and TF-8arm-PEG-DHA NPs for 24, 48 or 72 h. Analysis of *in vitro* cytotoxicity measurements showed that DHA (0.2 μg/mL) induced cell death, which was dependent upon length of incubation. As shown in Fig. 6a,b, the time-dependent cytotoxic effect of the TF-8arm-PEG-DHA NPs was evident, which indicated that 41.8% LLC and 62.7% A549 cells survival after 24 h, 23.4% LLC and 49.1% A549 survival after 48 h, only 19.2% LLC and 33.2% A549 survival after 72 h (the concentration equivalent to native DHA). As shown in Fig. 6c,d, the cell viability of 8arm-PEG-DHA NPs and TF-8arm-PEG-DHA NPs decreased with increasing DHA concentration, and there were no significant differences between the two nanoparticles to A549 cells. At the lowest concentration (0.2 μg/mL), 8arm-PEG-DHA NPs and TF-8arm-PEG-DHA NPs exhibited efficient anti-tumor effects. In particular, TF-8arm-PEG-DHA NPs showed the highest anti-tumor efficiency at all concentrations. This suggests that the TF-8arm-PEG-DHA NPs uptake by the cells is higher than 8arm-PEG-DHA NPs due to receptor-mediated endocytosis.

To compare the potency of nanoparticles, a drug concentration corresponding to 50% death of the cells (IC_50_) was estimated from survival curves in Fig. 6c,d, obtained from replicate experiments. The IC_50_ of 8arm-PEG-DHA NPs and TF-8arm-PEG-DHA NPs were slightly greater than free DHA (Table 2), and ranked as 8arm-PEG-DHA > TF-8armPEG-DHA > DHA. The IC_50_ of 8arm-PEG-DHA and TF-8arm-PEG-DHA complex stated in this article refer to DHA equivalents. For example, the IC_50_ of TF-8arm-PEG-DHA complex is 1.17μg/ml means that the concentration contains 1.17 μg/ml of DHA and 11.26 μg/ml the whole TF-8arm-PEG-DHA complex, here the loading of DHA in the TF-8arm-PEG-DHA complex is 10.39 wt%.

### Transferrin competition

**Figure 7:**
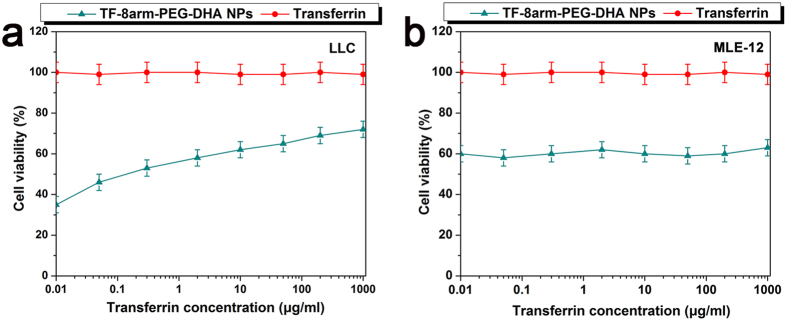
Effect of free transferrin on viability of LLC (**a**) and MLE-12 cells (**b**) incubated with TF-8arm-PEG–DHA NPs.

In order to further evaluate the role of transferrin in the cellular uptake of TF-8arm-PEG–DHA NPs, LLC cells and MLE-12 cells were employed as transferrin receptor (TFR) overexpressing cancer cells and TFR deficiency cancer cells, respectively. The cells were incubated with the nanoparticles (0.2 μg/mL) in culture medium containing increasing concentrations of free transferrin as described previously with minor modification. The cytotoxicity of TF-8arm-PEG–DHA NPs against LLC cells was inhibited by excess free transferrin, and the cell viability increased with increasing transferrin concentration (Fig. 7a). However, their cytotoxicity against MLE-12 cells did not change as a function of transferrin concentration (Fig. 7b). For instance, the cell viability of TF-8arm-PEG–DHA NPs against LLC cells was approximately 35.8% for TF-8arm-PEG–DHA NPs and free transferrin concentrations of 0.01 μg/mL, but it was about 68% in the presence of 100 μg/mL free transferrin. These findings suggest that free transferrin molecules prevent the cellular uptake of the nanoparticles by competitive binding to TFR on the cell surface.

### Cellular uptake

**Figure 8:**
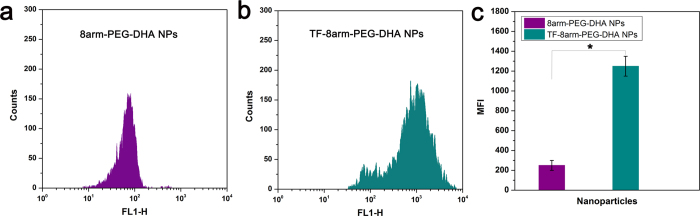
Flow cytometric analysis of LLC cells treated with 8arm-PEG–DHA NPs (**a**) and TF-8arm-PEG-DHA NPs (**b**).

The cellular uptake of FITC labeled NPs were evaluated using flow cytometry. The TF-modified NPs showed significantly enhanced cellular uptake compared to non-targeted NPs suggesting the importance of TF modification (Fig. 8a,b). As shown in Fig. 8c, the mean fluorescence intensity (MFI) of 8arm-PEG-DHA NPs was 251.05, however, TF-modified 8arm-PEG-DHA NPs was 1250.60 (~5 fold compared with 8arm-PEG-DHA NPs), indicating that cellular uptake of NPs was significantly increased after TF modification.

### Blood circulation of NPs after intravenous injection

**Figure 9:**
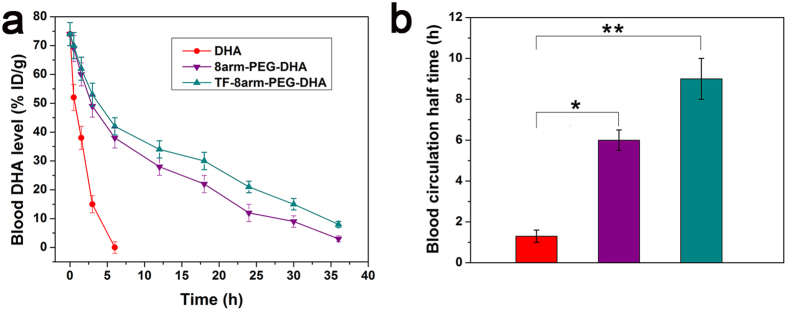
Blood circulation curves and half-time of 8arm-PEG–DHA NPs and TF-8arm-PEG–DHA NPs compared with free DHA (**a,b**). Error bars were based on six mice per group at each time point.

Longer blood circulation time can offer nanoparticles more opportunities to reach the tumor site, and thus enhanced the therapeutic effect. The determined drug concentration after hydrolysis under basic condition was actually the total DHA in plasma. The plasma clearance curves of free DHA and nanoparticles in mice were shown in Fig. 9a. Disappearance of DHA from the blood circulation after intravenous administration was very rapid with the plasma concentration below 20% of injected dose per gram (% ID/g) at 3 h. 8arm-PEG-DHA NPs removed a little slowly from the circulating system compared to free DHA, but the drugs were almost undetectable (8% ID/g) after 30 h. On the contrary, TF-8arm-PEG-DHA NPs exhibited a remarkable prolonged clearance with the drug levels of 16% ID/g at 30 h after administration. The half-time of free DHA in blood was only 1.3 h, while that of 8arm-PEG-DHA NPs, and TF-8armPEG-DHA NPs was significantly increased (5.9 h and 9.0 h, respectively), indicating that TF-8armPEG-DHA NPs could greatly prolong the circulation time in the blood (Fig. 9b).

### *In vivo* efficacy studies

**Figure 10:**
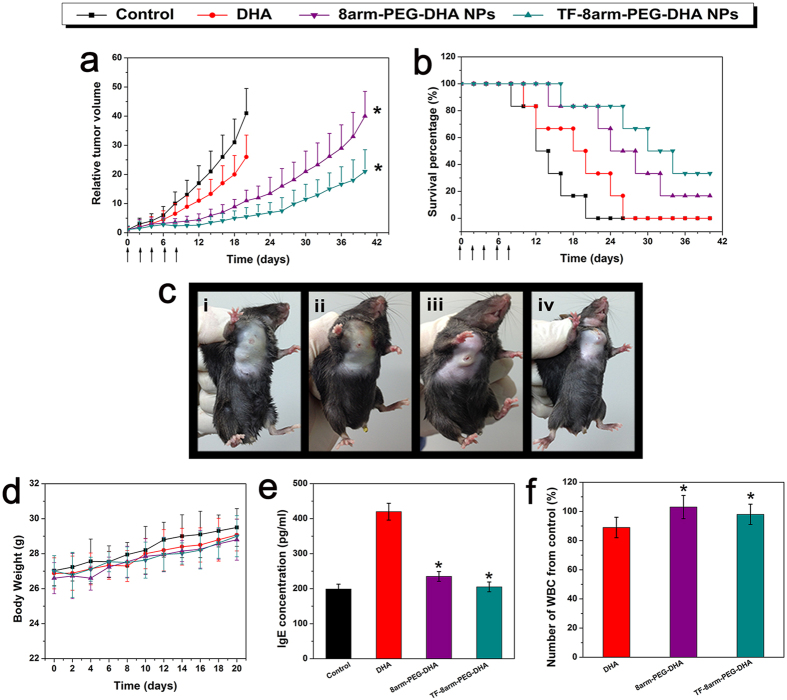
Anti-tumor efficacy of DHA, 8arm-PEG–DHA NPs and TF-8arm-PEG–DHA NPs in LLC-bearing mice model. (**a**) Tumor volumes of mice with different treatment groups. (**b**) Therapeutic efficacy of different treatments. (**c**) Tumor photographs from each treatment group excised on day 20 (i: control, ii: DHA, iii: 8arm-PEG–DHA NPs, and iv: TF-8arm-PEG–DHA NPs). (**d**) The body weight variation of mice with different treatments. (**e**) IgE levels of mice treated with different formulations for 30 min. (**f**) WBC change during different administrations in normal mice. Blood samples were collected from mice on day 2 after the last dosage treatment. Data are shown as mean ± SD; n = 6.

The results described above gave us great confidence to evaluate the anticancer effectiveness of nanoparticles in a mouse tumor model. During the treatment period, tumor growth of mice was not significantly inhibited by free DHA because of the poor solubility and bioavailability, as expected the tumor growth rates of mice treated with 8arm-PEG-DHA NPs and TF-8arm-PEG-DHA NPs were both slower than that of free DHA and control group, indicating favorable antitumor effects. Interestingly, TF-8arm- PEG-DHA NPs treatment exhibited greater tumor volume inhibition and survival benefit than 8arm-PEG-DHA NPs owing to the target ligand TF (Fig. 10a). As shown in Fig. 10b, the groups treated with free DHA and different nanoparticles showed varied levels of survival time and they were ranked as TF-8arm-PEG-DHA NPs > 8arm-PEG-DHA NPs > DHA, which was consistent with the results of tumor growth inhibition. The treatment with TF-8arm-PEG-DHA NPs resulted in 84.6% TGI (day 20) and 83.3% survival of animals (day 26). In contrast, the free DHA treatment resulted in 29.9% TGI (day 20) and 16.7% survival of animals (day 26) (Fig. 10a,b, Table 3). Importantly, in line with the literature, no signs of systemic toxicity were observed by monitoring general behavior, appetite and mice body weight (Fig. 10d). These results provide evidence that the combination of the PEG and the tumor cell targeting ligand of TF endowed TF-8arm-PEG-DHA NPs with significantly improvement in antitumor therapeutic efficacy.

### Evaluation of the side effects

Although the nanoparticles showed significant therapeutic effects *in vivo*, whether it had non-negligible adverse effects remained a critical issue. During the early development, type-1 hypersensitivity is the most common type of the hypersensitivity reaction. Some of the natural anticancer drugs, such as paclitaxel, docetaxel, and teniposide cyclosporine, were usually associated with a high incidence of type-1 hypersensitivity reaction. It has been demonstrated that IgE antibodies play an important part in mediating type-1 hypersensitivity responses. We thus selected IgE levels as the parameter for rapid evaluation of type-1 hypersensitivity reactions. The blood IgE levels of mice in different groups (DHA, 8arm-PEG-DHA NPs, and TF-8arm-PEG-DHA NPs) are shown in Fig. 10e. Mice treated with DHA displayed a higher IgE level than the PBS group, which might be ascribed to the poor solubility. As expected, no significant change of IgE level was observed in the 8arm-PEG-DHA NPs and TF-8arm-PEG-DHA NPs groups, which explored the idea that the use of nanoparticles could reduce the risk of hypersensitivity reactions substantially. The blood of mice after treatment with different groups were also collected to test the white blood cell (WBC) count, which is often used as an indicator of hematologic toxicity. The total WBC count of mice treated with DHA showed an obvious decrease over the normal group (Fig. 10f). No discernible decrease in WBC number of the mice treated with 8arm-PEG-DHA NPs and TF-8arm-PEG-DHA NPs were observed, indicating that the nanoparticles could avoid severe hematotoxicity.

## Discussion

Artemisinin has shown promise as novel anti-tumor drugs with high efficacy and low toxicity. Previous studies have indicated that artemisinin-based drugs could inhibit the growth of a variety of tumors. Dihydroartemisinin (DHA) is the most potent of artemisinin derivatives, it has stronger anti-malarial activity than its parent compound. Moreover, DHA has exhibited significant cytotoxicity to tumor cells. However, its clinical application has limited owe to low aqueous solubility and short circulation time.

To address the multiple challenges in high-performance delivery of insoluble anticancer agents, we have successfully prepared a novel nanocarrier TF-8arm-PEG-DHA NPs that synergistically holds many advantages. In terms of loading efficiency, 8arm-PEG was selected owe to its highly efficient incorporation of drugs compared to traditional liner PEG. In addition, the solubility of DHA was obvious increased due to the hydrophilic portion of PEG and TF. And after intravenous injection, these PEGylated nanoparticles could display their superior stealth ability, therefore, resulting in a prolonged circulation time. When encountering tumor tissue, they could also extravasate via the leaky vessels by the EPR effect and achieve active tumor targeting through specific interaction between TF and TFR.

The *in vitro* release study showed that the TF-8arm-PEG–DHA NPs displayed a preferential release under weakly acidic conditions (pH 4.5), which was benefit to the enhanced cytotoxicity. In addition, in the presence of esterase, which is abundant in cytoplasm, all the nanoparticles quickly hydrolyzed and released the DHA. The *in vitro* cytotoxic assay showed potent effect of TF-8arm-PEG-DHA NPs against LLC and A549 cells, there is only 19.2% LLC and 33.2% A549 cells survival after 72 h; however, it exists a little difference due to the relative difference in cell surface TFR expression levels and suggests a highly selective nature for tumor cells that overexpress TFRs.

Further *in vivo* studies demonstrated the favorable synergistic effect of PEG and TF, which benefited the achievement of better antitumor efficacy, higher survival rate and less side effects compared with free DHA. All these results together supported that the formulation developed in this work exhibited great potential as an effective tumor targeting delivery system for insoluble anticancer drugs.

In conclusion, a high drug-loading target nanoparticles, TF-8arm-PEG-DHA NPs, was successfully prepared with a hydrophobic anticancer core of DHA and a hydrophilic shell of PEG. The TF-8arm-PEG-DHA NPs showed high solubility in an aqueous solution, good stability and moderate particle size. In addition, *in vitro* cytotoxicity test demonstrated the excellent anticancer activities of TF-8arm-PEG-DHA NPs with potency similar to that of free DHA. The results of *in vivo* tumor growth inhibition studies confirmed the enhanced efficacy of TF-modified 8arm-PEG-DHA NPs to non-modified and free DHA with low toxicity. Therefore, TF-8arm-PEG-DHA NPs might be an effective delivery system for delivery of poorly soluble drugs used for the treatment of TFR-overexpressing carcinomas.

## Methods

### Synthesis of 8arm-PEG–DHA and TF-8arm-PEG–DHA conjugates

8arm-PEG–DHA conjugate. The 8arm-PEG–DHA conjugate was synthesized via esterification reaction by using EDC as a coupling reagent and DMAP as an organic catalyst in dichloromethane (DCM), as previously reported^39^. Briefly, 8arm-PEG-COOH (1.25 g, 0.125 mmol) and DHA (0.28 g, 1.0 mmol) were dissolved with 15 mL of dichloromethane (DCM). The solution was cooled to 0 °C and EDC (0.19 g, 1.0 mmol) and DMAP (0.12 g, 1.0 mmol) were added. The mixture was stirred at 0 °C for 1 h and at room temperature overnight. The solvent was evaporated under vacuum. The residue was dissolved in 25 mL of DCM, and the crude product was precipitated with ethyl ether (50 mL). After filtration, the resulting solids were recrystallized with a mixture of DMF/IPA (12 mL/48 mL). Then, the solids were filtered, washed with ethyl ether (2 × 50 mL), and dried under vacuum at 40 °C to give 8arm-PEG-DHA. The molar ratio results indicated that approximately 1 or 2 functional groups of 8arm-PEG–COOH remained unconjugated.

TF-8arm-PEG–DHA conjugate. 50 mg of 8arm-PEG-DHA conjugate was dissolved in 10 mL of phosphate buffer saline (PBS) solution. EDC (4 mg) and NHS (6 mg) were then added to the 8arm-PEG-DHA dissolved solution at room temperature for 15 min with gentle stirring. 8 mg TF was dissolved in 5 mL PBS solution. NHS-activated 8arm-PEG-DHA solution and TF solution were mixed and reacted with gentle stirring at room temperature for 6 h. After the reaction, the resulting solution was introduced into the dialysis membrane (molecular weight cut off, MWCO 30000 Da) and dialyzed against PBS solution to remove the unreacted reagents for 12 h. The total volume was adapted to 25 mL, and this solution was used to measure an *in vitro* and *in vivo* evaluation. In addition, the powder of TF-8arm-PEG-DHA conjugate was obtained by lyophilisation.

### Characterization of 8arm-PEG–DHA and TF-8arm-PEG–DHA conjugates

The 8arm-PEG-DHA and TF-8arm-PEG-DHA conjugates were characterized by proton nuclear magnetic resonance spectroscopy (^1^H-NMR) to confirm the successful synthesis. The ^1^H-NMR analyses were recorded on a Bruker DRX-600 Avance III spectrometer with deuterated chloroform (CDCl_3_) or deuterium oxide (D_2_O) as the solvent at room temperature. DHA, 8arm-PEG-DHA and TF-8arm-PEG-DHA (5 mg of each) was dissolved in 1 mL of CDCl_3_ or D_2_O, and the products were assessed using an NMR spectrometer.

The DHA amounts of the 8arm-PEG-DHA and TF-8arm-PEG-DHA were determined by HPLC (Agilent 1200, USA) in triplicate with UV detection at 210 nm. A C18-column (VYDAC 214TP54, 4.6 × 250 mm, Agilent 1200, USA) was used with a mobile phase consisted of water and acetonitrile (40:60 v/v) and the flow rate was 0.6 mL/min. The DHA concentrations in the samples were calculated from a calibration curve. The assay was linear over the tested concentration range. The mass and molar ratio of drug-to-carrier were calculated as mDHA/mConjug and nDHA/nConjug.

The solubility test of 8arm-PEG-DHA and TF-8arm-PEG-DHA against deionized water was examined using a UV spectrophotometer (VU1601, Shimazu, Japan)^40^. The concentrations of DHA, 8arm-PEG-DHA and TF-8arm-PEG-DHA were 10 mg/mL based on the amount of DHA.

### Preparation of nanoparticles

Nanoparticles were prepared by a precipitation method according to previous reports^41^. TF-8arm-PEG–DHA conjugate (0.20 g) dissolved in 0.2 mL of DMSO and added dropwise to 3.8 mL normal saline solution, which was then vortexed for 1 min. The resulting TF-8arm-PEG–DHA NPs solutions were transferred to a MWCO 3500 cartridge, and dialyzed against normal saline (100 mL) for 3 h with two exchanges of dialysate. The mean particle size and size distribution were determined by dynamic light scattering (DLS) with a particle analyzer (Zetasizer Nano-ZS, Malvern Instruments Ltd, Malvern, UK). The nanoparticls morphology was examined by transmission electron microscopy (TEM, JEOL JEM-2000 FX-II). 8arm-PEG–DHA NPs were prepared in a similar fashion as TF-8arm-PEG–DHA NPs.

### *In vitro* drug release

The release of DHA from the nanoparticles was analyzed by a dialysis method. 8arm-PEG–DHA NPs and TF-8arm-PEG–DHA NPs PBS solution (1 mg/mL, 5 mL) at pH 7.4 or 4.5 were loaded into a dialysis bag (MWCO 3500). Then the dialysis bag was immersed in 200 mL of PBS buffer (pH 7.4 or 4.5) at 37 °C with gentle agitation. PBS medium (1 mL) was withdrawn at timed intervals and the DHA concentration in the medium was determined by an Agilent 1200 (Agilent, USA) HPLC instrument. It employs a VYDAC 214TP54 (C18, 300A, 5 mm, 4.6 × 250 mm) with a UV detector, using a gradient of 15–100% of acetonitrile in 0.05% TFA at a flow rate of 1 mL/min. The release experiments were conducted in triplicate, and the results were expressed as mean ± standard deviation (SD). Esterase (30 units) was added into the dialysis bag when the DHA release in the presence of esterase was studied.

### *In vitro* cell cytotoxicity

Human lung cancer cells (A549) and murine lewis lung carcinoma (LLC) cells were obtained from the Peking University Health Science Center (Beijing, China) and were grown in the listed medium: A549 (RPMI 1640 with 10% FBS, 1% streptomycin–penicillin) and LLC (DMEM with 10% FBS, 1% streptomycin–penicillin). All cells were grown in a humidified incubator at 37 °C, 5% CO_2_.

The *in vitro* anticancer efficacy of the 8arm-PEG–DHA NPs and TF-8arm-PEG–DHA NPs against LLC and A549 cells were evaluated by a CCK-8 assay^42^. The specific experimental process was carried out as follows: approximate 2 × 10^3^ LLC cells/well and 4 × 10^3^ A549 cells/well were seeded in 96-well plates with 180 μL of DMEM medium and incubated overnight at 37 °C before the test. 8arm-PEG–DHA NPs and TF-8arm-PEG–DHA NPs were serially diluted in cell culture media and dissolved directly into media whereas free DHA was added from a stock solution in DMSO (1 mg/mL) and incubated for 24 h, 48 h and 72 h. At the end of the incubation period, 20 μL of CCK-8 solution was added to all wells of the plate and incubated for another 2 h at 37 °C. The percentage of cell viability can be calculated by measuring the absence of absorption by using a spectramax M5 at 450 nm. The half maximal inhibitory concentration (IC_50_) of the DHA and 8arm-PEG–DHA NPs and TF-8arm-PEG–DHA NPs were calculated using Origin^®^ 8.6 (OriginLab, Northampton, USA). The experiments were performed in triplicates and data was represented as mean ± SD.

### Cellular uptake study

For FCM analysis, the fluorescent probe, FITC, was loaded into the NPs for fluorescent labeling by a precipitation method. Briefly, FITC (1 mg) and 8arm-PEG-DHA conjugate (30 mg) were dissolved into 0.2 ml of DMSO and stirred for 15 min. The solution was then added to 3.8 ml of deionized water dropwise and stirred for 2 h. After stirring, the solution was loaded into a dialysis bag (MWCO 3500) and dialyzed against deionized water. TF-8arm-PEG–DHA/FITC NPs were prepared in a similar fashion as 8arm-PEG–DHA NPs FITC NPs.

In order to evaluate the cellular uptake of TF-8arm-PEG–DHA NPs, LLC cells at log phase were seeded onto a 6-well plate at a density of 3 × 10^5^ cells per well and cultured in 1 mL of medium. After 24 h, the media was removed, and cells were exposed to serum-free medium containing 8arm-PEG–DHA NPs (2.0 μg/ml, 2 ml) and TF-8arm-PEG–DHA NPs (1.17 μg/ml, 2 ml) for 4 h at 37 °C. The incubated cells were washed three times with cold PBS to eliminate trace product and detached with 0.02% EDTA–PBS and then suspended in PBS containing 0.1% BSA. The suspended cells were directly introduced to a FACSort flow cytometer (Becton Dickinson, USA).

### Blood circulation of different nanoparticles after intravenous injection

The normal mice were injected with DHA, 8arm-PEG-DHA NPs and TF-8arm-PEG-DHA NPs through the tail vein. After intravenous administration, blood samples were collected at different time from the orbital plexus and centrifuged immediately at 3,000 rpm for 10 min at 37 °C. To determine the level of total DHA in each plasma sample, 100 μL of plasma was mixed with 50 μL of 0.1 N HCl for 15 min in water bath at 37 °C, allowing the hydrolysis of the nanoparticles. After that, 0.1 N NaOH (50 μL) was added, followed by 100 μL methanol. After vortexed for 2 min, the mixture was sonicated for 5 min and centrifuged at 5,000 rpm for 5 min. The clear supernatant was dried under nitrogen, reconstituted by 100 μL methanol before HPLC analysis. The HPLC employs a VYDAC 214TP54 (C18, 5 mm, 4.6 × 250 mm) with a UV detector, using a gradient of 60% of acetonitrile in 0.05% TFA at a flow rate of 0.6 mL/min. Blood circulation data were plotted as the blood DHA or DHA nanoparticles levels with the unit of percentage of injected dose per gram (% ID/g) against time after injection.

### *In vivo* efficacy studies

All animal experiments were performed in accordance with Guide for the Care and Use of Laboratory Animals, and approved by Beijing Administration Office of Laboratory Animal. Subcutaneous tumor xenograft models were established in the right axillary flank region of female C57BL/6 mice (6–7 weeks) by injecting 1 × 10^6^ LLC cells per mouse and the mice were randomly divided into four groups. The mice of different groups were injected intravenously with PBS, DHA, 8arm-PEG–DHA NPs and TF-8arm-PEG–DHA NPs (the dose equal to DHA) at one day intervals (n = 5, 10 mg/kg) when tumors reached an average volume of 100 to 150 mm^3^. It is important to note that the doses or concentrations of 8arm-PEG–DHA NPs and TF-8arm-PEG–DHA NPs stated in this article refer to DHA equivalents. In the observation phase, mice were monitored for tumor sizes and body weights every other day. The tumor volume was calculated using the formula: (L × W^2^)/2, where L is the longest and W is the shortest tumor diameter (millimeter). The relative tumor volume (RTV) was calculated at each measurement time point (where RTV was equal to the tumor volume at a given time point divided by the tumor volume prior to the initial treatment). For efficacy studies, the percentage of tumor growth inhibition (%TGI) was calculated using the following formula: [(C − T)/C] × 100%, where C is the mean tumor volume of the control group at a specified time and T is the mean tumor volume of the treatment group at the same time. To monitor potential toxicity, the weight of each mouse was measured. For humane reasons, animals were killed and regarded as dead if the implanted tumor volume reached 5000 mm^3^ or at the end of the experiment (>6 weeks).

### Evaluation of hypersensitivity

To evaluate allergic reaction, four groups of tumor bearing mice with weight of 26–28 g (n = 6) were injected with physiological saline, DHA, 8arm-PEG-DHA NPs, and TF-8arm-PEG-DHA NPs every two days at a DHA dose of 10 mg/kg, respectively. After being injected for 10 days, the orbit blood of mice in different groups was collected and centrifuged. Serum samples were analyzed according to the procedure of mouse IgE ELISA using a hematology analyzer (MEK-7222K, Nihon Kohden Celltac E). To further evaluate the hematological toxicity of different groups, 200 μL of blood of each mouse was collected to test the white blood cell (WBC) number after final administration by a blood cell analyzer (MEK-7222K, Japan).

### Statistical analysis

All data in this study were reported as the mean ± SD unless otherwise illustrated. The statistical analysis was measured using ANOVA. P** < **0.05 was considered to be statistically significant for evaluating the differences between the treatment groups.

## Additional Information

**How to cite this article**: Liu, K. *et al*. Self-assembled targeted nanoparticles based on transferrin-modified eight-arm-polyethylene glycol–dihydroartemisinin conjugate. *Sci. Rep*. **6**, 29461; doi: 10.1038/srep29461 (2016).

## Figures and Tables

**Table 1:** Drug loading efficiency, solubility, hydrolysis and particle size of nanoparticles.

Compound	Mass ratio	Molar ratio	Solubility (T%)^a^	Hydrolysis _t1/2_ (h)^b^		Size (nm)
				pH 7.4	Ph 4.5	
DHA	–	–	0.95	–	–	–
8arm-PEG-DHA NPs	15.12 ± 1.23	6.26 ± 0.52	90.24	126.2	65.5	112.42 ± 17.28
TF-8arm-PEG-DHA NPs	10.39 ± 1.02	93.39 ± 7.56	96.70	121.3	54.4	147.64 ± 21.36

^a^Equivalent to native DHA.

^b^Based on the release of DHA.

**Table 2:** *In Vitro* Cytotoxicity analysis (IC_50_, μg/ml).

Compound	LLC	A549
DHA	0.73 ± 0.012	0.63 ± 0.017
8-arm-PEG-DHA	2.01 ± 0.053	2.55 ± 0.059
TF-8arm-PEG-DHA	1.17 ± 0.041	1.95 ± 0.047

**Table 3:** LLC Xenograft Model (50 mg/kg single dose): Efficacy Comparison.

Compound	Mean TV ± SD (mm^3^)^a^	RTV^a^	TGI (%)^a^	Cures (%)^b^
Control	4650 ± 980	38.4 ± 12.2	–	0
DHA	3159 ± 653	25.3 ± 8.4	29.9 ± 8.4	16.7
8arm-PEG-DHA NPs	1115 ± 257	10.4 ± 3.9	76.3 ± 15.2	66.7
TF-8armPEG-DHA NPs	720 ± 190	6.5 ± 3.2	82.8 ± 20.4	83.3

^a^Mean tumor volume (TV), RTV, and % TGI data were taken at day 20. (By day 20, a significant percentage of control animals were euthanized due to excess tumor burden.).

^b^% cures were taken at day 26.

## References

[b1] Brannon-Peppas L. & Blanchette J. O. Nanoparticle and targeted systems for cancer therapy. Advanced drug delivery reviews. 56, 1649–1659 (2004). 15350294 10.1016/j.addr.2004.02.014

[b2] Klayman D. L. Qinghaosu (artemisinin): an antimalarial drug from China. Science. 228, 1049–1055 (1985). 3887571 10.1126/science.3887571

[b3] O’Neill P. M. & Posner G. H. A medicinal chemistry perspective on artemisinin and related endoperoxides. Journal of medicinal chemistry. 47, 2945–2964 (2004). 15163175 10.1021/jm030571c

[b4] Dell’Eva R. . Inhibition of angiogenesis *in vivo* and growth of Kaposi’s sarcoma xenograft tumors by the anti-malarial artesunate. Biochemical pharmacology. 68, 2359–2366 (2004). 15548382 10.1016/j.bcp.2004.08.021

[b5] Singh N. P. & Lai H. C. Artemisinin induces apoptosis in human cancer cells. Anticancer research. 24, 2277–2280 (2004). 15330172

[b6] Efferth T. Molecular pharmacology and pharmacogenomics of artemisinin and its derivatives in cancer cells. Current drug targets. 7, 407–421 (2006). 16611029 10.2174/138945006776359412

[b7] Lai H., Sasaki T., Singh N. P. & Messay A. Effects of artemisinin-tagged holotransferrin on cancer cells. Life sciences. 76, 1267–1279 (2005). 15642597 10.1016/j.lfs.2004.08.020

[b8] AlKadi H. O. Antimalarial drug toxicity: a review. Chemotherapy. 53, 385–391 (2007). 17934257 10.1159/000109767

[b9] Chen H., Sun B., Pan S., Jiang H. & Sun X. Dihydroartemisinin inhibits growth of pancreatic cancer cells *in vitro* and *in vivo*. Anti-cancer drugs. 20, 131–140 (2009). 19209030 10.1097/CAD.0b013e3283212ade

[b10] Hou J., Wang D., Zhang R. & Wang H. Experimental therapy of hepatoma with artemisinin and its derivatives: *in vitro* and *in vivo* activity, chemosensitization, and mechanisms of action. Clinical cancer research. 14, 5519–5530 (2008). 18765544 10.1158/1078-0432.CCR-08-0197

[b11] Brouwers J., Brewster M. E. & Augustijns P. Supersaturating drug delivery systems: The answer to solubility‐limited oral bioavailability? Journal of pharmaceutical sciences. 98, 2549–2572 (2009). 19373886 10.1002/jps.21650

[b12] Kolate A. . PEG—a versatile conjugating ligand for drugs and drug delivery systems. Journal of controlled release. 192, 67–81 (2014). 24997275 10.1016/j.jconrel.2014.06.046

[b13] Pasut G. & Veronese F. Polymer–drug conjugation, recent achievements and general strategies. Progress in polymer science. 32, 933–961 (2007).

[b14] Acharya S. & Sahoo S. K. PLGA nanoparticles containing various anticancer agents and tumour delivery by EPR effect. Advanced drug delivery reviews. 63, 170–183 (2011). 20965219 10.1016/j.addr.2010.10.008

[b15] Maeda H., Bharate G. & Daruwalla J. Polymeric drugs for efficient tumor-targeted drug delivery based on EPR-effect. European journal of pharmaceutics and biopharmaceutics. 71, 409–419 (2009). 19070661 10.1016/j.ejpb.2008.11.010

[b16] Knop K., Hoogenboom R., Fischer D. & Schubert U. S. Poly (ethylene glycol) in drug delivery: pros and cons as well as potential alternatives. Angewandte chemie international edition. 49, 6288–6308 (2010). 10.1002/anie.200902672 20648499

[b17] Ashley G. W., Henise J., Reid R. & Santi D. V. Hydrogel drug delivery system with predictable and tunable drug release and degradation rates. Proceedings of the national academy of sciences. 110, 2318–2323 (2013). 10.1073/pnas.1215498110 PMC3568318 23345437

[b18] Greenwald R. B., Choe Y. H., McGuire J. & Conover C. D. Effective drug delivery by PEGylated drug conjugates. Advanced drug delivery reviews. 55, 217–250 (2003). 12564978 10.1016/s0169-409x(02)00180-1

[b19] Liu K.-f. . Design, synthesis and *in vivo* antitumor efficacy of novel eight-arm-polyethylene glycol–pterostilbene prodrugs. RSC advances. 5, 51592–51599 (2015).

[b20] Ahmad I., Longenecker M., Samuel J. & Allen T. M. Antibody-targeted delivery of doxorubicin entrapped in sterically stabilized liposomes can eradicate lung cancer in mice. Cancer research. 53, 1484–1488 (1993). 8453612

[b21] Kirpotin D. B. . Antibody targeting of long-circulating lipidic nanoparticles does not increase tumor localization but does increase internalization in animal models. Cancer research. 66, 6732–6740 (2006). 16818648 10.1158/0008-5472.CAN-05-4199

[b22] Bagalkot V., Farokhzad O. C., Langer R. & Jon S. An Aptamer–Doxorubicin Physical Conjugate as a Novel Targeted Drug‐Delivery Platform. Angewandte chemie international edition. 45, 8149–8152 (2006). 10.1002/anie.200602251 17099918

[b23] Pangburn T. O., Petersen M. A., Waybrant B., Adil M. M. & Kokkoli E. Peptide-and aptamer-functionalized nanovectors for targeted delivery of therapeutics. Journal of biomechanical engineering. 131, 074005 (2009). 19655996 10.1115/1.3160763

[b24] Kato Y. & Sugiyama Y. Targeted delivery of peptides, proteins, and genes by receptor-mediated endocytosis. Critical Reviews™ in Therapeutic drug carrier systems. 14, (1997). 9282268

[b25] Bies C., Lehr C.-M. & Woodley J. F. Lectin-mediated drug targeting: history and applications. Advanced drug delivery reviews. 56, 425–435 (2004). 14969751 10.1016/j.addr.2003.10.030

[b26] Leamon C. P. & Low P. S. Folate-mediated targeting: from diagnostics to drug and gene delivery. Drug discovery today. 6, 44–51 (2001). 11165172 10.1016/s1359-6446(00)01594-4

[b27] Sudimack J. & Lee R. J. Targeted drug delivery via the folate receptor. Advanced drug delivery reviews. 41, 147–162 (2000). 10699311 10.1016/s0169-409x(99)00062-9

[b28] Dufès C., Al Robaian M. & Somani S. Transferrin and the transferrin receptor for the targeted delivery of therapeutic agents to the brain and cancer cells. Therapeutic delivery. 4, 629–640 (2013). 23647279 10.4155/tde.13.21

[b29] Huang R.-Q. . Efficient gene delivery targeted to the brain using a transferrin-conjugated polyethyleneglycol-modified polyamidoamine dendrimer. The FASEB journal. 21, 1117–1125 (2007). 17218540 10.1096/fj.06-7380com

[b30] Qian Z. M., Li H., Sun H. & Ho K. Targeted drug delivery via the transferrin receptor-mediated endocytosis pathway. Pharmacological reviews. 54, 561–587 (2002). 12429868 10.1124/pr.54.4.561

[b31] Joneli J. . Determination of carbohydrate-deficient transferrin in human serum by capillary zone electrophoresis: Evaluation of assay performance and quality assurance over a 10-year period in the routine arena. Electrophoresis. 34, 1563–1571 (2013). 23512316 10.1002/elps.201200653

[b32] Sawant R. R. . Targeted transferrin-modified polymeric micelles: enhanced efficacy *in vitro* and *in vivo* in ovarian carcinoma. Molecular pharmaceutics. 11, 375–381 (2013). 24325630 10.1021/mp300633f

[b33] Xu C.-C. . Synthesis and *in vitro* antitumor evaluation of dihydroartemisinin-cinnamic acid ester derivatives. European journal of medicinal chemistry. 107, 192–203 (2016). 26595184 10.1016/j.ejmech.2015.11.003

[b34] Nam J.-P. . Evaluation of polyethylene glycol-conjugated novel polymeric anti-tumor drug for cancer therapy. Colloids and surfaces B: biointerfaces. 120, 168–175 (2014). 24918700 10.1016/j.colsurfb.2014.04.013

[b35] Ku H.-K. . Interpretation of protein quantitation using the Bradford assay: comparison with two calculation models. Analytical biochemistry. 434, 178–180 (2013). 23201266 10.1016/j.ab.2012.10.045

[b36] Wang D. . Ternary system of dihydroartemisinin with hydroxypropyl-β-cyclodextrin and lecithin: simultaneous enhancement of drug solubility and stability in aqueous solutions. Journal of pharmaceutical and biomedical analysis. 83, 141–148 (2013). 23732534 10.1016/j.jpba.2013.05.001

[b37] Wang J., Tian S., Petros R. A., Napier M. E. & DeSimone J. M. The complex role of multivalency in nanoparticles targeting the transferrin receptor for cancer therapies. Journal of the American chemical society. 132, 11306–11313 (2010). 20698697 10.1021/ja1043177 PMC2923393

[b38] Shen Y. . Prodrugs forming high drug loading multifunctional nanocapsules for intracellular cancer drug delivery. Journal of the American Chemical Society. 132, 4259–4265 (2010). 20218672 10.1021/ja909475m

[b39] Dai L. . Novel multiarm polyethylene glycol-dihydroartemisinin conjugates enhancing therapeutic efficacy in non-small-cell lung cancer. Scientific reports. 4 (2014). 10.1038/srep05871 PMC5376196 25070490

[b40] Madhaiyan K., Sridhar R., Sundarrajan S., Venugopal J. R. & Ramakrishna S. Vitamin B 12 loaded polycaprolactone nanofibers: a novel transdermal route for the water soluble energy supplement delivery. International journal of pharmaceutics. 444, 70–76 (2013). 23370432 10.1016/j.ijpharm.2013.01.040

[b41] Ernsting M. J., Tang W.-L., MacCallum N. & Li S.-D. Synthetic modification of carboxymethylcellulose and use thereof to prepare a nanoparticle forming conjugate of docetaxel for enhanced cytotoxicity against cancer cells. Bioconjugate chemistry. 22, 2474–2486 (2011). 22014112 10.1021/bc200284b

[b42] Wang L.-L., Hu R.-C., Dai A.-G. & Tan S.-X. Bevacizumab induces A549 cell apoptosis through the mechanism of endoplasmic reticulum stress *in vitro*. International journal of clinical and experimental pathology. 8, 5291 (2015). 26191230 PMC4503101

